# Hydrochlorate of Cocaine

**Published:** 1885-01

**Authors:** J. M. Hull

**Affiliations:** Augusta, Ga.; Special Professor of Diseases of the Eye and Throat in the Medical College of Georgia, Medical Department of the University of Georgia, Member of the Augusta Academy of Medicine, etc.


### HYDROCHLORATE OF COCAINE.

### REPORT OF FOURTEEN CASES OPERATED UPON UNDER ITS INFLUENCE.

By J. M. HULL, M. D.,

Special Professor of Diseases of the Eye and Throat in the Medical College of Georgia, Medical Department of the University of Georgia, Member of the Augusta Academy of Medicine, etc.

After procuring a 2%. solution of the hydrochlorate of Cocaine, on November 16th last, I proceeded to test its merits, and, having had such perfect success, I now give my experience.

The first case was one of opacity of cornea, in a gentleman 43 years of age, the result of penetrating injury to it, and in cicatrix of which the iris was detained. Supersensitiveness existed. In order to detach the iris a solution of atropia, grs. iv. to ℥i. was instilled into the eye and maximum dilatation speedily obtained. Five minutes later two drops of a 2% solution of the Cocaine was dropped into the eye; two minutes later the patient complained of a dull, sleepy feeling, and five minutes after this there was such perfect anæsthesia the operation of scraping the cornea was performed without the patient's being aware of it.

The same day I had the pleasure of applying the same solution to an eye containing a piece of steel in the cornea, and removed the foreign body six minutes later without pain.

On two subsequent occasions foreign bodies were removed five minutes after making a single application with same solution, with same result.

In a case of double pterygium two applications were made of two drops each of the solution, three minutes apart, and operated five minutes after the second application, discovering anæsthesia and a marked hæmostatic influence.

A third pterygium operation was performed with the drug with like success.

Desiring to see to what depth the anæsthesia extended, I applied two drops every three minutes until three applications had been made, to the inner surface of the upper lid, over a chalazion, in a boy ten years old. From the incision he experienced no pain, and, while in the later steps of removal there was some sensibility, it was so slight as to make the anæsthesia for this class of operations satisfactory.

Three days later I removed a mucous cyst from lower lip of same boy, after making three applications, with a like experience. In these operations the hæmostatic properties were also marked. In a case of extreme symblepheron, the result of a burn, I obtained absolute anæsthesia after using two drops of solution three times, every three minutes.

With a view of determining whether the drug would be useful in operations involving the deeper tissues, I performed an iridectomy. In making the corneal incision there was no pain, but upon removal of iris there was pain.

Believing that a better result for such operations could be obtained from a 4% solution, I procured it, and found that, after dropping two drops into the eye every three minutes until three applications were made, and operating at the expiration of five minutes from last application, iridectomies, and even cataract extraction, could be made without the slightest sensation.

A case of strabismus was also operated, without pain, by using the 2% solution twice.

From the above 14 cases the following deductions are reached:

1st. Perfect local anæsthesia can be obtained for all operations involving the conjunctiva and cornea from a 2% solution of Hydrochlorate of Cocaine, two drops of which must be dropped into the eye every three minutes until three applications are made.

2d. For operations involving the deeper tissues a 4% solution should be used in the same way.

3d. Hydrochlorate of Cocaine is an undoubted hæmostatic, producing anæmia of the mucous surfaces.

